# Coinfection with *Strongyloides* and SARS-CoV-2: A Systematic Review

**DOI:** 10.3390/tropicalmed8050248

**Published:** 2023-04-25

**Authors:** Elena C. Rosca, Carl Heneghan, Elizabeth A. Spencer, Annette Plüddemann, Susanna Maltoni, Sara Gandini, Igho J. Onakpoya, David Evans, John M. Conly, Tom Jefferson

**Affiliations:** 1 Department of Neurology, Victor Babes University of Medicine and Pharmacy, Piata Eftimie Murgu 2, 300041 Timisoara, Romania; 2 Centre for Evidence Based Medicine, Nuffield Department of Primary Care Health Sciences, University of Oxford, Radcliffe Observatory Quarter, Oxford OX2 6GG, UK; carl.heneghan@phc.ox.ac.uk (C.H.); elizabeth.spencer@phc.ox.ac.uk (E.A.S.); annette.pluddemann@phc.ox.ac.uk (A.P.); 3 Division of Research and Innovation, IRCCS Azienda Ospedaliero-Universitaria di Bologna, 40138 Bologna, Italy; susanna.maltoni@gmail.com; 4 Department of Experimental Oncology, IEO European Institute of Oncology IRCCS, 20141 Milan, Italy; sara.gandini@ieo.it; 5 Department of Continuing Education, University of Oxford, Rewley House, 1 Wellington Square, Oxford OX1 2JA, UK; igho.onakpoya@conted.ox.ac.uk (I.J.O.); jefferson.tom@gmail.com (T.J.); 6 Li Ka Shing Institute of Virology and Department of Medical Microbiology & Immunology, University of Alberta, Edmonton, AB T6G 2E1, Canada; devans@ualberta.ca; 7 Departments of Medicine, Microbiology, Immunology & Infectious Diseases, and Pathology & Laboratory Medicine, Synder Institute for Chronic Diseases and O’Brien Institute for Public Health, Cumming School of Medicine, University of Calgary and Alberta Health Services, Calgary, AB T2N 1N4, Canada; john.conly@albertahealthservices.ca

**Keywords:** SARS-CoV-2, COVID-19, coinfection, *Strongyloides*, systematic review

## Abstract

Background: Treatments for COVID-19, including steroids, might exacerbate *Strongyloides* disease in patients with coinfection. We aimed to systematically review clinical and laboratory features of SARS-CoV-2 and *Strongyloides* coinfection, investigate possible interventions, assess outcomes, and identify research gaps requiring further attention. Methods: We searched two electronic databases, LitCOVID and WHO, up to August 2022, including SARS-CoV-2 and *Strongyloides* coinfection studies. We adapted the World Health Organization—Uppsala Monitoring Centre (WHO-UMC) system for standardized case causality assessment to evaluate if using corticosteroids or other immunosuppressive drugs in COVID-19 patients determined acute manifestations of strongyloidiasis. Results: We included 16 studies reporting 25 cases of *Strongyloides* and SARS-CoV-2 coinfection: 4 with hyperinfection syndrome; 2 with disseminated strongyloidiasis; 3 with cutaneous reactivation of strongyloidiasis; 3 with isolated digestive symptoms; and 2 with solely eosinophilia, without clinical manifestations. Eleven patients were asymptomatic regarding strongyloidiasis. Eosinopenia or normal eosinophil count was reported in 58.3% of patients with *Strongyloides* reactivation. Steroids were given to 18/21 (85.7%) cases. A total of 4 patients (19.1%) received tocilizumab and/or Anakirna in addition to steroids. Moreover, 2 patients (9.5%) did not receive any COVID-19 treatment. The causal relationship between *Strongyloides* reactivation and COVID-19 treatments was considered certain (4% of cases), probable (20% of patients), and possible (20% of patients). For 8% of cases, it was considered unlikely that COVID-19 treatment was associated with strongyloidiasis reactivations; the relationship between the *Strongyloides* infection and administration of COVID-19 treatment was unassessable/unclassifiable in 48% of cases. Of 13 assessable cases, 11 (84.6%) were considered to be causally associated with *Strongyloides*, ranging from certain to possible. Conclusions: Further research is needed to assess the frequency and risk of *Strongyloides* reactivation in SARS-CoV-2 infection. Our limited data using causality assessment supports recommendations that clinicians should screen and treat for *Strongyloides* infection in patients with coinfection who receive immunosuppressive COVID-19 therapies. In addition, the male gender and older age (over 50 years) may be predisposing factors for *Strongyloides* reactivation. Standardized guidelines should be developed for reporting future research.

## 1. Introduction

Strongyloidiasis is a parasitic disease caused by *Strongyloides stercoralis*, a soil-transmitted nematode (roundworm). It has been reported in tropical and subtropical regions and areas of low endemicity in temperate climates [1]. It is estimated that 600 million people are infected worldwide [2], but accurate data on the prevalence are lacking in endemic countries. The unique features of *Strongyloides* are their ability to persist and replicate within a host for decades while determining minimal or no symptoms and their potential to cause life-threatening disease by dissemination and hyperinfection in the setting of immunosuppression [3,4].

Patients with strongyloidiasis present a broad spectrum of clinical manifestations, with five main clinical pictures: (1) asymptomatic intestinal infection; (2) acute infection with cutaneous manifestations and Loeffler’s syndrome; (3) chronic intestinal disease with chronic anemia, eosinophilia, malabsorption, and chronic diarrhea; (4) hyperinfection syndrome (HS); and (5) disseminated strongyloidiasis (DS) [5,6].

Most infected people are asymptomatic or experience intermittent symptoms, mainly digestive manifestations (from mild abdominal pain or diarrhea to more severe presentations mimicking inflammatory bowel disease), respiratory signs (cough, wheezing, asthma, and chronic bronchitis), and dermatological signs (rash and pruritus). Infected persons may also present with systemic involvement, e.g., weight loss and cachexia [4]. Immunocompromised patients have an increased risk of developing HS and DS, which may be fatal [4]. The use of corticosteroids is the most common trigger for HS [7,8], the syndrome being documented even after short courses of corticotherapy (e.g., 4 days) [9,10] and in low doses (e.g., 20 mg of prednisone/day) [11]. Other risk factors for HS and DS comprise immunosuppressive treatment, hematopoietic stem cell and solid organ transplantation, HIV/AIDS, HTLV-1 infection, hematologic malignancies (e.g., leukemia and lymphoma) or solid tumors, collagen vascular disease, chronic renal failure, and the use of histamine H2-receptor antagonists and antacids [5,12,13,14,15].

The Coronavirus Disease 2019 (COVID-19) has affected over 750 million people worldwide, with almost 6.8 million deaths [16]. Consequently, a high number of patients coinfected with both *Strongyloides* and SARS-CoV-2 is to be anticipated.

Current therapies in patients with moderate or severe SARS-CoV-2 infection include administering anti-inflammatory agents with immunosuppressive effects, such as dexamethasone and tocilizumab [17]. Therefore, patients with coinfection have a potential risk of developing HS and DS [17,18,19,20], as immunosuppression can worsen the parasitic disease [17]^.^

In this context, there is a need to investigate coinfection with S*trongyloides* and SARS-CoV-2, as HS and DS may be associated with mortality rates up to 90% if untreated [21,22]. In addition, there are still knowledge gaps concerning several aspects of this coinfection.

Our objectives were to systematically review the clinical and laboratory features of SARS-CoV-2 and *Strongyloides* coinfection and to investigate potential interventions and outcomes. Additionally, we aimed to identify the research gaps that require further attention. If the data exists, recommendations could be made for screening in high-risk settings.

## 2. Materials and Methods

We conducted searches in the following electronic databases: LitCOVID and the World Health Organization (WHO) COVID-19 (which cover PubMed, MEDLINE, Web of Science, EMBASE, MedRxiv, and other databases) up to 23 August 2022. As these databases are specific for COVID-19, we used the following terms: “*Strongyloides*,” “Strongyloidiasis,” “Anguillulose,” and “Anguillulosis”; a search string to identify articles on SARS-CoV-2 infection was not necessary. We looked for additional research through searches of the reference lists of relevant articles. We did not set language restrictions. All references were stored, organized, and managed with bibliographic software (EndNote 20, Clarivate Analytics, Philadelphia, PA, USA) [23].

We included studies reporting on patients with concomitant acute SARS-CoV-2 infection and strongyloidiasis without age, gender, or region restrictions. We included case reports, case series, and prospective or retrospective, observational or interventional studies. Besides primary studies, we planned to include systematic reviews if available. Conference abstracts were included if the authors did not publish a full article on the study [23].

From the included studies, we extracted the following information: publication details (authors, year, and country), type of study, patient data (age, gender, origin country, immunological status, comorbidities, and medication), clinical signs and symptoms, the time between SARS-CoV-2 infection and strongyloidiasis manifestations and diagnosis, paraclinical findings, postmortem investigations, treatment, COVID-19 severity, evolution and outcome of coinfection, and the assumed mechanism of manifestations of strongyloidiasis [23].

One reviewer (ECR) extracted data from the included studies, and these were independently checked by a second reviewer (EAS).

We adapted the World Health Organization—Uppsala Monitoring Centre (WHO-UMC) system for standardized case causality assessment [24] to evaluate if using corticosteroids in COVID-19 patients determined the acute manifestations of strongyloidiasis. We made a combined assessment accounting for the clinical, paraclinical, and pharmacologic aspects of the case history and the quality of the documentation of observations. The causality categories included certain, probable/likely, possible, unlikely, or unclear associations between using steroids and strongyloidiasis [23]. One primary reviewer (ECR) assessed the causality in the included studies, and a second reviewer (JMC) independently evaluated the causality to assess for concordance of the causality categorization. Any disagreements were resolved via consensus. Where possible, we reported the results of individual patients. We considered meta-analyses inappropriate, as the included studies presented substantial heterogeneity.

## 3. Results

**Figure 1:**
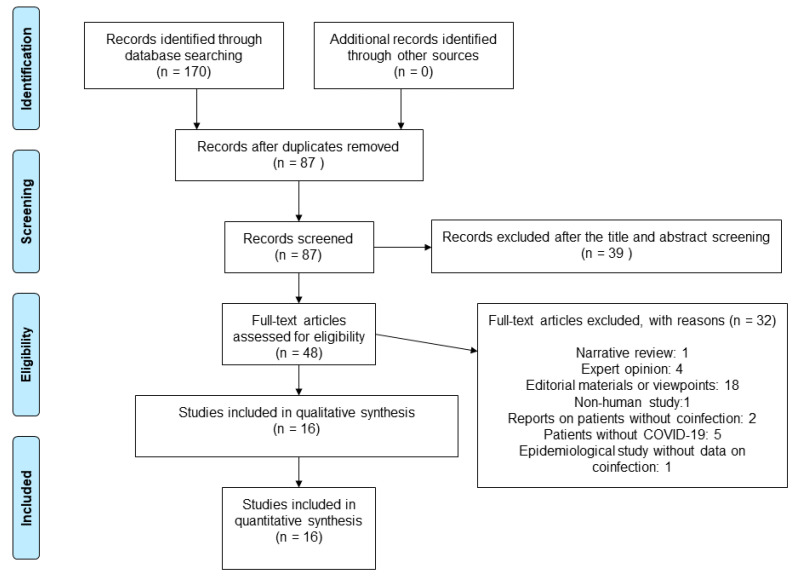
Flow diagram showing the process for inclusion of studies investigating coinfection with *Strongyloides* and SARS-CoV-2.

Our searches identified 170 articles, of which 48 were considered potentially eligible (Figure 1).

We assessed full texts of 48 studies, out of which 32 were excluded: 1 narrative review, 4 expert recommendations, 18 editorial materials or viewpoints, 1 non-human study, 2 reports on patients without coinfection, 5 articles on patients without COVID-19, and 1 epidemiological study without data on coinfection (Supplementary Material S1). In total, we included 16 studies reporting 25 cases of coinfection (Supplementary Material S2) [25,26,27,28,29,30,31,32,33,34,35,36,37,38,39,40]. The detailed characteristics of the included studies are presented in Supplementary Material S3.

The included studies were conducted in Spain [27,31,33,35,38], Italy [32], Belgium [40], Ireland [36], the USA [29,30], Iran [25,26], India [28,39], and Peru [34]; one meeting abstract did not report the country of the authors [37]. Of the 25 included cases, 4 were from Ecuador [30,35,38,40], 3 from Bolivia [27,31], 3 from Honduras [27,31], 2 from Iran [25,26], 2 from India [28,39], 2 from Peru [31,34], 1 from Colombia [31], 1 from Cambodia [29], 1 from Morocco [31], 1 from Italy [32], 1 from Nigeria [36], and 1 from Nicaragua [37]. One study with three cases reported that the patients were from Latin America [33].

The years of publication ranged from 2020 through 2022. One article reported on a retrospective, longitudinal, descriptive study aiming to evaluate all patients admitted with a COVID-19 diagnosis at a tertiary care hospital [31]. Another article reported a retrospective observational study conducted in a tertiary-level hospital, including all COVID-19 patients from *Strongyloides* endemic areas and treated with prophylactic ivermectin [33]. The rest of the articles were case reports.

Among the 22 cases with information on age and gender, 11 (50%) were females, and 11 (50%) were males, with ages ranging from 4 years to 74 years.

### 3.1. Hyperinfection Syndrome (HS) and Disseminated Strongyloidiasis (DS)

Among 25 cases of coinfection, 4 patients developed HS [26,28,29,36]. Three cases received corticosteroids [26,28,29], and one patient did not receive any treatment for COVID-19, as he was asymptomatic [36]. In addition, in one case, the patient was administered baricitinib and remdesivir [29]. Two patients were reported to present DS [30,37]. Both received corticotherapy, and one of the patients was also treated with tocilizumab [30]. Only 1 patient presented an unfavorable outcome, with death despite treatment with oral ivermectin (200 μg/kg for 14 days) [29].

### 3.2. Cutaneous Reactivation of Strongyloidiasis

Three patients were reported with cutaneous reactivation of chronic *S. stercoralis* infection [27,35]. They all received dexamethasone for 7–12 days. The skin lesions appeared after 7–10 days of treatment, with resolution within 48 h of treatment with ivermectin [30,35].

### 3.3. Gastrointestinal Manifestations of Strongyloidiasis

Three cases presented gastrointestinal symptoms [32,38,39]. One male patient developed abdominal pain and itching on the twenty-fifth day of hospitalization [32]. His SARS-CoV-2 infection was treated with dexamethasone, hydroxychloroquine, lopinavir/ritonavir, and two doses of tocilizumab [32]. Another male patient developed abdominal pain two months after COVID-19 onset [38]. He received methylprednisolone, tocilizumab, and anakinra while hospitalized for the SARS-CoV-2 infection, and his symptoms were resolved. He had a normal eosinophil count throughout the initial hospital stay but presented with eosinophilia upon a second hospitalization for *Strongyloides* reactivation. The gastrointestinal symptoms were remitted after albendazole, but the eosinophilia persisted; therefore, he also received ivermectin [38]. The third case with gastrointestinal symptoms was treated for SARS-CoV-2 infection with oral methylprednisolone, hydroxychloroquine, favipiravir/remedesvir, aztreonam, and azithromycin [39]. However, he presented with symptoms of an acute abdomen at admission, and investigations revealed ascariasis and strongyloidiasis [39]. All patients improved after antiparasitic treatment.

### 3.4. Isolated Eosinophilia

Two patients with SARS-CoV-2 infection were reported to have only eosinophilia, without clinical signs of strongyloidiasis [31,40]. Both received corticosteroids for COVID-19; one patient also received interleukin-1 receptor antagonist therapy with anakinra [40]. In the latter case, eosinophilia was absent at initial admission (before the start of systemic corticosteroid treatment), during the stay in the intensive care unit, and under corticosteroid treatment. Follow-up investigations (after day 49) showed a rising number of blood eosinophils, the highest value being 2670 eosinophils/mL. After one week, a single dose of ivermectin led to a marked eosinophilia [40].

### 3.5. Asymptomatic Strongyloidiasis

Eleven patients did not present any manifestations of strongyloidiasis after coinfection with SARS-CoV-2 [25,31,33,34].

In a retrospective, longitudinal, descriptive study, Lorenzo et al. reported six patients with COVID-19 and chronic strongyloidiasis [31]. Among them, two received dexamethasone, three were treated with hydroxychloroquine, lopinavir/ritonavir, and azithromycin, and one patient had no specific medications for the SARS-CoV-2 infection. According to the authors, none of the patients developed symptomatic strongyloidiasis or eosinophilia [31].

Another retrospective study reported on the prophylactic administration of ivermectin in COVID-19 patients from endemic areas treated with corticosteroids or other immunosuppressive drugs [33]. Among 35 patients from endemic areas, 83% received dexamethasone (6 mg/24 h), 14% methylprednisolone bolus (250 mg), 12% tocilizumab (400 mg), and 3% did not have any immunosuppressive treatment. Only 3 (9%) individuals had positive serology for *S. stercoralis*; they did not develop HS or DS. Furthermore, no patient had eosinophilia, but the authors did not specify if any of them received previous treatment for *Strongyloides* [33].

Alian et al. report on the case of a patient diagnosed with mucormycosis one month after COVID-19 [25]. Upon admission, he received dexamethasone and remdesivir. However, on the third day of admission, the RT-PCR test for SARS-CoV-2 infection was negative, and the COVID-19 medication was discontinued. The patient died 32 days after admission. The authors consider that the patient did not present any clinical signs of strongyloidiasis, attributing the clinical picture to the fungal infection. However, *Strongyloides* were reported in the stool analyses, but no information on any antiparasitic treatment was provided [25].

Nakandakari et al. documented the case of a four-year-old child with skin lesions, acute abdominal pain, and episodes of upper gastrointestinal bleeding [34]. He presented with dry cough and rhinorrhea eight days before admission, followed by fever and epigastric pain. The IgM and IgG for SARS-CoV-2 were positive. He received corticotherapy, metronidazole, and ivermectin to resolve symptoms. The authors concluded that the patient presented IgA vasculitis in the context of SARS-CoV-2 infection. They considered the possibility that symptoms were due to strongyloidiasis as improbable [34].

### 3.6. Laboratory Investigations

The diagnosis of SARS-CoV-2 infection was based on positive RT-PCR in 14/25 (56%) patients, but the Cycle threshold (Ct) is not specified in any case [29,30,31,32,37,38,39,40]. In one child, the diagnosis was based on IgM and IgG positivity [34]. The diagnostic test was not specified in 10 cases [25,26,27,28,33,35,36].

The *Strongyloides* reactivation was diagnosed based on clinical and laboratory findings and a history of travel or living in an endemic country. The authors performed stool analysis for 9/25 (36%) patients [25,28,29,32,34,35,37,38,39]. Agar plate results were available for two patients [28,35]. Serology was performed in 18/25 (72%) individuals [27,29,30,31,33,35,36,38,40], and examination of bronchoalveolar lavage [29,37] or sputum [30] was conducted in 3/25 (12%) patients. Only 1 case (4%) had a positive immunofluorescence antibody test (IFAT) [32], and 1 case (4%) had a positive RT-PCR [40]. Histopathological examination was performed in 2/25 (8%) patients [26,36].

Among 12 patients with *Strongyloides* reactivation, 7 (58.33%) presented initially with normal eosinophils count or eosinopenia [27,28,29,30,38,40]. A total of 3 patients (25%) were reported to have eosinophilia [26,32,37]. Nonetheless, in these latter cases, 1 was 3 weeks after COVID-19 illness [26], another case was on his 25th day after SARS-CoV-2 infection diagnosis [32], and in the 3rd patient, the authors did not specify the day of illness when the blood examination was performed [37]. The level of eosinophils was not reported in 2 patients (16.67%) [35,36].

In patients with *Strongyloides* reactivation, the lymphocyte level was within the normal range in 3/12 (25%) cases [26,27,30], and 2/12 (16.67%) had lymphocytopenia [29,38]. In 7/12 (58.33%) cases, the authors do not provide information on the lymphocyte level [27,28,32,35,36,37,40].

### 3.7. Time Interval between SARS-CoV-2 Infection and Strongyloides Manifestations

In patients with HS or DS, the clinical signs of strongyloidiasis manifested between 14–30 days after COVID-19 diagnosis [26,28,29,30]. However, one patient was considered asymptomatic for SARS-CoV-2 infection [36]. The cutaneous reactivation of strongyloidiasis appeared between 8–20 days after COVID-19 diagnosis [27,35]. The patients with solely gastrointestinal strongyloidiasis manifestations presented symptoms 25–60 days after viral infection diagnosis [32,38], and 1 patient with isolated eosinophilia presented 59 days after a COVID-19 diagnosis [40].

### 3.8. Strongyloidiasis Treatment and Outcomes

Seven patients received ivermectin; six cases improved [27,32,35,36,37,40], but one patient with HS died [29]. Three patients with positive *S. stercoralis* serology received ivermectin prophylactic treatment; they did not develop HS or DS [32]. Six patients were treated with a combination of albendazole and ivermectin, with favorable outcomes [26,28,30,38,39]. The authors do not mention any specific antiparasitic treatment for 7 patients with positive serology; they had a complete clinical recovery, being hospitalized for COVID-19 between 4 and 20 days [31]. In addition, one pediatric patient was treated with metronidazole and ivermectin, with favorable evolution; however, the authors reported he had IgA vasculitis [34]. In one paper, the authors do not provide any information on the strongyloidiasis treatment; the patient also presented with mucormycosis and died 32 days after admission [25].

The main characteristics of the included patients are presented in Table 1.

### 3.9. Case Causality Assessment

We evaluated the causality between the clinical manifestations of *Strongyloides* reactivation and the use of corticosteroids or immunosuppressive agents in patients with COVID-19. The evaluation was enhanced by the use of two independent reviewers, one of whom has considerable experience with strongyloidiasis. We considered a certain causality relationship in 1/25 (4%) of cases [30], a probable causality relationship in 5/25 (20%) of patients [26,27,29,38], and a possible relationship for 5/25 (20%) cases [28,32,35,37,40]. It was considered unlikely that the COVID-19 treatment caused strongyloidiasis symptoms in 2/25 (8%) cases [34,36]. In addition, we considered that the relationship between the *Strongyloides* infection and the use of corticosteroids or other immunosuppressive drugs was unassessable/unclassifiable in 12/25 (48%) individuals, as the information provided by authors was incomplete or contradictory and could not be complemented or verified [25,31,33,39]. Of the 13 cases which were assessable 11 (84.6%) were considered to be causally associated with COVID-19 treatment, ranging from possible to certain (Table 2).

## 4. Discussion

We identified 16 studies reporting 25 cases of *Strongyloides* and SARS-CoV-2 coinfection. The evidence suggests that HS and DS may be present in patients with coinfection, but the exact mechanism is unclear. Among the cases of coinfection, four patients presented HS [26,28,29,36], two had DS [30,37], three developed a cutaneous reactivation of *Strongyloides* infection [27,35], three cases presented with isolated gastrointestinal symptoms [32,38,39], and two individuals presented solely with eosinophilia [30,40], without clinical manifestations. Eleven patients were asymptomatic with regard to strongyloidiasis. Nonetheless, 3/11 cases received prophylactic ivermectin [33], 1/11 was reported to present with mucormycosis [25], and 1/11 was diagnosed with IgA vasculitis in the context of SARS-CoV-2 infection [34].

In all the cases, the authors do not provide evidence of *Strongyloides* infection prior to COVID-19. Hypothetically, there is the possibility that the patients were infected with *Strongyloides* after the SARS-CoV-2 infection, and the parasite reactivation is not due to the use of steroids. Nonetheless, 8/25 patients presented symptoms of strongyloidiasis reactivation during the hospitalization for COVID-19 [27,30,32,35,37,39,40]. Other 6/25 patients developed reinfection symptoms in a short interval after hospitalization for SARS-CoV-2 infection [26,28,29,38] ranging from 6 days to 4 weeks, or immediately after COVID-19 diagnosis [31,36], while the incubation period for *Strongyloides* infection ranges between 2 to 4 weeks. The other 9/25 patients did not present any signs of *Strongyloides* reactivation [25,31,33,34], and the parasitosis was diagnosed at the time of SARS-CoV-2 infection.

Furthermore, the diagnosis of SARS-CoV-2 infection was made in 14/26 (53.8%) patients based on positive RT-PCR, but without data on Ct, and based on IgM and IgG positivity in 1/26 cases. Therefore, in 10/26 (38.5%) cases, the certainty of SARS-CoV-2 infection is hampered by the lack of information on the diagnostic methods.

Regarding the treatment received for the SARS-CoV-2 infection, the authors reported individual data for 21 patients. Steroid therapy was prescribed for 18/21 (85.71%) [26,27,28,29,30,31,32,35,37,38,39,40]: 3/18 (16.67%) developed HS [26,28,29], 2/18 (11.11%) presented DS [30,37], 3/18 (16.27%) had cutaneous reactivation of strongyloidiasis [27,35], 3/18 (16.27%) complained of exclusively gastrointestinal symptoms [32,38,39], and 2/18 (11.11%) presented with isolated eosinophilia [30,40]. Additionally, 4 patients (19.05%) received immunosuppressive drugs; 2 individuals were treated with tocilizumab and developed DS [30] and gastrointestinal symptoms [38]. Anakinra was prescribed in two cases: one presented isolated eosinophilia [40], and one had digestive complications [38]. Nonetheless, the patients receiving tocilizumab or anakinra also received corticotherapy; therefore, it is challenging to ascertain the role of the immunosuppressants in developing strongyloidiasis. Only 2 patients (9.52%) did not receive any COVID-19 treatment. Interestingly, one case developed DS [36], and one had no symptoms of strongyloidiasis or eosinophilia [31].

When assessing the causality between the clinical manifestations of *Strongyloides* and the use of corticosteroids or immunosuppressive agents, we considered that there was a certain causality relationship only in 4% of cases. A probable relationship was found in 20% of patients, and a possible relationship in 20%.

Several factors might have contributed to these findings. It is well-established that corticosteroids increase the risk of *Strongyloides* reactivation. Furthermore, in immunosuppressed patients with hematological malignancies, corticosteroids were a predictive factor for strongyloidiasis with an odds ratio (OR) of 2.29 [41]. The mechanisms by which corticosteroids increase the susceptibility to severe Strongyloidiasis are not entirely elucidated. Most authors hypothesize that corticotherapy predisposes to reactivation of the parasite through its immunosuppressive effects, mainly their effects on eosinophils, which are essential mediators of the immune response to *Strongyloides* larvae [42]. Additionally, some researchers suggested that corticotherapy may directly affect the parasites, precipitating their dissemination [43].

Interestingly, HS and DS have also been reported in other viral infections, such as HIV or HTLV-1 [4,6,10]. For example, in patients with *Strongyloides* and HIV coinfection, the researchers found a significant inverse correlation between the survival rate, the CD4+ T-cell counts, and peripheral eosinophilia [44]. Additionally, in advanced HIV stages, patients present a variety of abnormalities in the regulation of cytokine expression, including an increase in Th-2 cytokines and a reduction in the level of Th-1 cytokines [45]. The increased risk of HTLV1-infected patients to severe manifestations of strongyloidiasis highlights the importance of T cell response in the host’s defense against the parasite. HTLV-1 causes both a Th-1 response and the expansion of regulatory T cells, resulting in a decreased production of IL-5 and depletion of eosinophils [46,47]. In addition, HTLV-1 may favor the *Strongyloides* activation by decreasing IgE responses and causing a switch from Th2 to Th1 immunological response [47], which will lead to a synergic effect with corticosteroid therapy [10]. Some authors even consider strongyloidiasis an opportunistic infection [48], specifically in the patients with impaired immunity presenting an increased *S stercoralis* load.

COVID-19 may induce immunological alterations, including lymphocytopenia, thrombocytopenia, and eosinopenia. For example, 50–70% of patients hospitalized for SARS-CoV-2 infection presented a decreased eosinophil count [49,50]. Additionally, corticosteroids may induce lymphocytopenia. In the present review, only four patients were reported with lymphocytopenia [25,29,38,39]; two developed severe strongyloidiasis symptoms, and two cases presented other confounding factors like coinfection with Ascaris species or mucormycosis. Notably, most authors did not report on the lymphocyte count. Eosinopenia or a normal eosinophil count was reported in 58.3% of patients with *Strongyloides* reactivation, including the case of a patient with chronic eosinophilia that remitted during COVID-19, with rising eosinophils during recovery.

Eosinophils play a cornerstone role in the antihelminthic host defense [51,52,53]. Eosinophilia is encountered in 70% of patients with chronic strongyloidiasis but only 20% of HS patients. Additionally, eosinopenia was reported to be associated with a worse outcome in HS [53,54]. However, it remains unclear if eosinophils are directly involved in the pathophysiology of organ failures or if they represent just a bystander of an appropriate antiparasitic immune response [53].

The clinical picture of HS and DS comprises various manifestations, the most frequent being fever, gastrointestinal symptoms, and respiratory symptoms, including respiratory failure [4,53]. Hence, it is difficult to distinguish the symptoms due to SARS-CoV-2 infection and the severe manifestations of strongyloidiasis. The mimicry bias might be present, especially in patients without eosinophilia, with a high risk of misclassification.

A literature review on HS reported that the median time from corticosteroid treatment initiation to the manifestation of HS symptoms was 42 days (IQR 14–90) [53]. Other authors report that the mean daily dosage of corticosteroid in patients with severe strongyloidiasis was 52 ± 42 (mean ± SD) mg of prednisolone equivalent, with a duration of therapy ranging from 4 days to 20 years [10]. We found that in *S. stercoralis* and SARS-CoV-2 coinfection, the patients presented with strongyloidiasis symptoms much earlier after the COVID-19 diagnosis: 8 to 30 days for HS and DS, and 25 to 60 days for patients with solely digestive manifestations. In addition, we found one case report of HS in a patient without any treatment for COVID-19 [36]. Therefore, it is reasonable to assume that SARS-CoV-2 infection may favor *Strongyloides* reactivation leading to a pro-*Strongyloides* synergy with immunosuppressive therapy. Furthermore, in other patients receiving corticotherapy, reactivation of *Strongyloides* is seldom or never reported (e.g., myasthenia gravis [55,56], multiple sclerosis). Additionally, the reverse could be true; namely, the presence of coinfection with a similar clinical presentation could cause an overestimation of symptoms due to *S. stercoralis*.

In addition, although it is well known that eosinophilia is present in many chronic strongyloidiasis cases, it is possible that due to SARS-CoV-2 infection itself or the treatments employed, pre-existing eosinophilia may be masked. Therefore, a high suspicion index is necessary to detect the parasitic infection and diagnose the severe complications of *S. stercoralis*.

Regarding the age of patients, there is the possibility that older patients are at higher risk of Strongyloides reactivation. In our review, all patients with HS, DS, and isolated digestive symptoms were over 50 years (see Table 1). On the contrary, among the patients without reactivation, 6/9 (66.7%) were < 50 years [31,34]. In addition, among patients with isolated eosinophilia, 1 was 33 years old [31], and 1 was 59 years [40]. Furthermore, among 6 patients with severe strongyloidiasis manifestations (HS and DS), 5 (83.3%) were males [26,28,29,30,37]. The only female who developed HS received no steroid therapy [36]. A predominance of the male gender was also observed in patients with cutaneous reactivation, with 66.7% of patients being males [27,32,35]. Isolated eosinophilia was reported in one male [40] and one female [31]. In contrast, among patients without Strongyloides reactivation, 5/7 (71.4%) were females [31,34].

Some recommendations advocate screening for *S. stercoralis* in patients with a high risk of exposure, in all patients with immunosuppression, and in candidates for immunosuppressive treatment [57]^.^ Furthermore, if an appropriate diagnostic test is unavailable, specific treatment with ivermectin should be pre-emptively provided in immunosuppressed patients and candidates for immunosuppression [57]. Additionally, in the context of coinfection with SARS-CoV-2, it is essential to recognize and screen patients at risk of strongyloidiasis [17]. Currently, in patients with *S. stercoralis* and SARS-CoV-2 coinfection, who are under consideration to receive specific COVID-19 therapies that alter the immune response and may lead to HS or DS, clinicians should screen and treat for *Strongyloides* infection [17,22]. However, some authors raised concerns about the applicability of immunosuppressive treatment in COVID-19 patients and the risk of harm rather than benefits from steroid administration as a consequence of the different epidemiology of other infectious diseases, such as tuberculosis or strongyloidiasis, which may be reactivated or worsened [58].

Data on the epidemiology of the coinfection remains scarce. A study found that, among 227 cases with strongyloidiasis and SARS-CoV-2 coinfection, 4 individuals developed HS, with the death of 1 patient [59]. Nonetheless, the authors provide no specific data on the diagnosis, clinical picture, or treatment of the patients. Additionally, using specific diagnostic tests with low sensitivity would lead to an underestimation of the prevalence of *Strongyloides* reactivation.

The limitations of this present review are mainly related to the quality of the included studies. The finding of several cases of disseminated strongyloidiasis probably represents a reporting bias for severe disease, and thus, we may have missed less severe manifestations of strongyloidiasis. In addition, the data extraction was challenging due to missing, incomplete, or unclear descriptions of the information. This could be due to the need for standardized methodology and clear reporting criteria contributing to substantial methodological variation in SARS-CoV-2 studies. We also could not determine the contribution of COVID-19 disease to *Strongyloides* infections, independently from the COVID-19 treatment-associated immunosuppression, that would naturally place the individuals at risk for re-activation of untreated strongyloidiasis, or the effect of pre-existing strongyloidiasis on the severity of COVID-19. Other factors that may induce bias include the multiple variables that should be considered, from diagnosis to clinical and paraclinical findings, treatment, and outcomes.

Our findings emphasize the need for a standardized approach to investigation and reporting on strongyloidiasis and SARS-CoV-2 coinfection. Future studies should aim for a comprehensive assessment of patients. Factors that may influence the evolution or the diagnosis, such as the baseline characteristics of the patients, evolution, and treatments, should be consistently assessed across studies. Additionally, further epidemiological studies are needed.

## 5. Conclusions

Although the clinical presentation of strongyloidiasis has been well characterized, coinfection with SARS-CoV-2 may influence the clinical spectrum. Given the global incidence of SARS-CoV-2, including the endemic areas for *S. stercoralis*, a proportion of all *S. stercoralis* infections occur concurrently with infections by one or multiple pathogens. The association between strongyloidiasis and SARS-CoV-2 remains inadequately investigated, with several uncertainties regarding the epidemiology and clinical and paraclinical spectrum of the coinfection. Further research is needed to assess whether SARS-CoV-2 infection favors *Strongyloides* reactivation with a pro-*Strongyloides* synergy when combined with immunosuppressive therapy. Our limited data using causality assessment would support recommendations that clinicians should screen and treat for Strongyloides infection in patients with *S. stercoralis* and SARS-CoV-2 coinfection who receive specific COVID-19 therapies that are immunosuppressive. Furthermore, the male gender and older age (over 50 years) may be predisposing factors for *Strongyloides* reactivation. Additionally, standardized guidelines for reporting future research in this area should be developed.

## Figures and Tables

**Table 1:** The characteristics of included cases.

Study	Age	Gender	Underlying Diseases	*Strongyloides* Infection Manifestation	*Strongyloides* Infection Diagnosis	COVID-19 Diagnosis	Laboratory Findings	COVID-19 Treatment	*Strongyloides* Treatment	Outcome
Alian 2022 [25]	73	Female	Chronic kidney disease, diabetes mellitus, hypertension, dyslipidemia.	Mucormycosis. No strongyloidiasis manifestations.	Stool analysis	N/R	Lymphocytes decreased	Dexamethasone (8 mg daily) Remdesevir.	N/R	Death
Babazadeh 2022 [26]	70	Male	Mitral valve replacement, atrial fibrillation, heart failure.	HS	Histopathologic examination (gastric and duodenal mucosae)	N/R	Lymphocytes normal Eosinophils increased	Dexamethasone (6 mg/day iv, 10 days).	Ivermectin (200 μg/kg for 7 days) Albendazole (400 mg every 12 h for 10 days)	Remission of symptoms
Feria 2022, case 1 [27]	44	Male	Smoking, hypertension, obesity.	Cutaneous reactivation	Serology for *S. stercoralis* IgG (ELISA)	N/R	Eosinophils normal	Dexamethasone (6 mg/day, 7 days).	Ivermectin (200 mcg/kg/day for 2 days)	Resolution of the skin condition
Feria 2022, case 2 [27]	74	Female	Pyrazolone allergy, hypertension, dyslipidemia, chronic kidney disease, disseminated tuberculosis correctly treated.	Cutaneous reactivation	Serology for S. stercoralis IgG (ELISA)	N/R	Lymphocytes normal Eosinophils decreased	Dexamethasone (6 mg/day, 10 days).	Ivermectin (200 mcg/kg/day for 2 days)	Resolution of the skin condition
Gautam 2021 [28]	53	Male	N/R	HS	Stool microscopic examination Koga agar plate	N/R	Eosinophils normal	Methylprednisolone (60 mg iv, twice a day for 5 days).	Ivermectin Albendazole	Recovery
Kim 2022 [29]	63	Male	Diabetes mellitus, alcohol use disorder.	HS	Microscopic examination of the bronchoalveolar lavage fluid Serology for *S. stercoralis* IgG Stool microscopic examination	RT-PCR	Lymphocytes decreased Eosinophiles decreased	Dexamethasone (6 mg/d for 10 days); Baricitinib (10 mg/day, for 5 days) Remdesivir (100 mg/day, for 5 days).	Oral ivermectin (200 μg/kg for 14 days)	Death
Lier 2020 [30]	68	Male	Hypertension, diabetes mellitus complicated by peripheral neuropathy.	DS	Sputum culture Gram and iodine stains *Strongyloides* serum antibody Stool analysis	RT-PCR	Lymphocytes normal Eosinophils decreased	Methylprednisolone (40 mg iv every 8 h, 3 courses) Tocilizumab (once, iv at 8 mg/kg) Hydroxychloroquine (400 mg oral twice daily loading dose, then 200 mg oral twice daily for 5 days).	Ivermectin (200 μg/kg daily). Albendazole (400 mg orally every 12 h, a 2-week course	Improvement. Transferred to a skilled nursing facility
Lorenzo 2022, Patient 1 [31]	37	Female	Obesity, diabetes mellitus, dyslipidemia, previous strongyloidiasis (digestive symptoms, no cutaneous manifestations).	None	Serology for *S. stercoralis* IgG (ELISA)	RT-PCR	Eosinophils normal	Dexamethasone (6 mg once daily for 10 days).	N/R	Recovery
Lorenzo 2022, Patient 2 [31]	47	Female	Chagas disease, previous strongyloidiasis (digestive symptoms, asthma, no cutaneous manifestations).	None	Serology for *S. stercoralis* IgG (ELISA)	RT-PCR	Eosinophils normal	Dexamethasone (6 mg once daily, for 10 days) Remdesivir.	N/R	Recovery
Lorenzo 2022, Patient 3 [31]	33	Female	Previous strongyloidiasis (digestive symptoms, asthma, no cutaneous manifestations).	Eosinophilia	Serology for *S. stercoralis* IgG (ELISA)	RT-PCR	Eosinophils increased	Dexamethasone (6 mg once daily, for 10 days) Remdesivir.	N/R	Recovery
Lorenzo 2022, Patient 4 [31]	38	Male	Previous strongyloidiasis (asthma, no cutaneous manifestations).	None	Serology for *S. stercoralis* IgG (ELISA)	RT-PCR	Eosinophils normal	Azithromycin Hydroxychloroquine Lopinavir/ritonavir.	N/R	Recovery
Lorenzo 2022, Patient 5 [31]	22	Male	Crohn disease, previous strongyloidiasis (digestive symptoms, no cutaneous manifestations).	None	Serology for *S. stercoralis* IgG (ELISA)	RT-PCR	Eosinophils normal	Azithromycin Hydroxychloroquine Lopinavir/ritonavir.	N/R	Recovery
Lorenzo 2022, Patient 6 [31]	69	Female	Trigeminal neuralgia.	None	Serology for *S. stercoralis* IgG (ELISA)	RT-PCR	Eosinophils normal	Azithromycin Hydroxychloroquine Lopinavir/ritonavir.	N/R	Recovery
Lorenzo 2022, Patient 7 [31]	27	Female	Vitiligo, previous strongyloidiasis (digestive symptoms, no cutaneous manifestations).	None	Serology for *S. stercoralis* IgG (ELISA)	RT-PCR	Eosinophils normal	None.	N/R	Recovery
Marchese 2021 [32]	59	Female	Still’s disease, hypertension, repeated episodes of diffuse itching in the last 10 years, treated with topical steroids with partial improvement.	Digestive symptoms.	Stool examination IFAT serology	RT-PCR	Eosinophils increased	Hydroxychloroquine Lopinavir/ritonavir Dexamethasone (20 mg/day for 5 days, followed by 10 mg/day for other 6 days) Tocilizumab 8 mg/kg, 2 doses, 12 h apart.	Ivermectin (200 mcg/kg, oral, 4 days)	Improvement
Martinez 2021, 3/35 (9%) cases [33]	Average: 42.84 ± 11.38 years (among 35 patients)	52% of women (among 35 patients)	N/R	None	Serology for *S. stercoralis*	N/R	Eosinophils normal	Among 35 cases: 83% dexamethasone 6 mg/24 h, 14% methylprednisolone bolus 250 mg, 12% tocilizumab 400 mg, and 3% no immunosuppressive treatment.	Prophylactic: ivermectin 6 mg/8 h for 2 days.	No *Strongyloides* infection manifestations
Nakandakaria 2021 [34]	4	Female	The parents were tested positive and treated for COVID-19 a month earlier.	None	Stool examination	COVID-19 Rapid Test (IgM, IgG)	Eosinophils increased	N/R	Ivermectin (1 drop/kg/day for 2 days) Metronidazole (40 mg/kg/day every 8 h).	Recovery
Nunez-Gomez 2021 [35]	45	Male	Several episodes of suspected allergic reactions with rash and angioedema. The last episode occurred in 2018, and the trigger remained undetermined.	Cutaneous reactivation	Serology for *S. stercoralis* Stool Mueller-Hinton agar plate culture	N/R	N/R	Dexamethasone (6 mg/day, for 12 days)	Ivermectin (200 mg/kg, for 14 days).	Improvement
O’Dowling 2022 [36]	60	Female	No significant past medical history.	HS	Serology for *S. stercoralis* Pathological analysis of the small bowel specimen	N/R	N/R	None	Ivermectin (2 doses).	Improvement
Patel 2021 [37]	72	Male	N/R	DS	Stool microscopic examination Bronchoalveolar lavage Gram-stain	RT-PCR	Eosinophils increased	Dexamethasone	Ivermectin.	Improvement
Pintos-Pascual 2021 [38]	70	Male	Hypertension	Digestive reactivation	Fresh stool analysis Serological test	RT-PCR	Lymphocytes decreased Eosinophils normal	Methylprednisolone (250 mg boluses, for 5 days, followed by dose tapering, ending treatment at one month) Tocilizumab (day 6 to day 13). Anakinra (on days 10–13 and 19–24).	Albendazole (400 mg/12 h for 3 days) Ivermectin.	Resolution of symptoms
Singh 2021 [39]	58	Male	Diabetes, rheumatoid arthritis.	Unclear. Ascariasis—*S. stercoralis* coinfection	Stool microscopic examination	RT-PCR	Lymphocytes decreased Eosinophils increased	Methylprednisolone Aztreonam Hydroxychloroquine Favipiravir/remedesvir Azithromycin	Ivermectin (200 μg/kg/day, 2 weeks) Albendazole (400 mg every 12 h, 2 weeks)—for Ascariasis coinfection.	Improvement
Stylemans 2021 [40]	59	Male	Diabetes, smoking, chronic eosinophilia for 7 years.	Eosinophilia	Serology for *S. stercoralis* Molecular diagnosis of *S. stercoralis* in fresh fecal samples using RT-PCR	RT-PCR	Eosinophils normal	Anakinra Methylprednisolone (80 mg, tapered over 1 month; from day 49 quick tapering from 16 mg to stop over 7 days).	Ivermectin (single dose)	Recovery

Notes: Hyperinfection syndrome: HS; disseminated strongyloidiasis: DS; reverse transcription polymerase chain reaction: RT-PCR. Not reported: N/R.

**Table 2:** Causality assessment evaluating if the use of corticosteroids in COVID-19 patients determined the acute strongyloidiasis manifestations.

Study	Certain	Probable/Likely	Possible	Unlikely	Conditional/Unclassified	Unassessable/Unclassifiable
Alian 2022 [25]	No	No	No	No	No	Yes
Babazadeh 2022 [26]	No	Yes	No	No	No	No
Feria 2022 case 1 [27]	No	Yes	No	No	No	No
Feria 2022 case 2 [27]	No	Yes	No	No	No	No
Gautam 2021 [28]	No	No	Yes	No	No	No
Kim 2022 [29]	No	Yes	No	No	No	No
Lier 2022 [30]	Yes	No	No	No	No	No
Lorenzo 2022 patient 1 [31]	No	No	No	No	No	Yes
Lorenzo 2022 patient 2 [31]	No	No	No	No	No	Yes
Lorenzo 2022 patient 3 [31]	No	No	No	No	No	Yes
Lorenzo 2022 patient 4 [31]	No	No	No	No	No	Yes
Lorenzo 2022 patient 5 [31]	No	No	No	No	No	Yes
Lorenzo 2022 patient 6 [31]	No	No	No	No	No	Yes
Lorenzo 2022 patient 7 [31]	No	No	No	No	No	Yes
Marchese 2021 [32]	No	No	Yes	No	No	No
Martinez 2021 [33]	No	No	No	No	No	Yes
Nakandakaria 2021 [34]	No	No	No	Yes	No	No
Nunez-Gomez 2021 [35]	No	No	Yes	No	No	No
O’Dowling 2022 [36]	No	No	No	Yes	No	No
Patel 2021 [37]	No	No	Yes	No	No	No
Pintos-Pascual 2021 [38]	No	Yes	No	No	No	No
Singh 2021 [39]	No	No	No	No	No	Yes
Stylemans 2021 [40]	No	No	Yes	No	No	No

## Data Availability

All data included in this review are provided in the tables or the supplemental files.

## Supplementary Materials

The following supporting information can be downloaded at: https://www.mdpi.com/article/10.3390/tropicalmed8050248/s1, Supplemental Material S1. List of excluded studies; Supplemental Material S2. List of included studies. Supplemental Material S3. Characteristics of included studies.

## References

[1] McKenna M.L., McAtee S., Bryan P.E., Jeun R., Ward T., Kraus J., Bottazzi M.E., Hotez P.J., Flowers C.C., Mejia R. (2017). Human Intestinal Parasite Burden and Poor Sanitation in Rural Alabama. Am. J. Trop. Med. Hyg..

[2] Buonfrate D., Bisanzio D., Giorli G., Odermatt P., Fürst T., Greenaway C., French M., Reithinger R., Gobbi F., Montresor A. (2020). The Global Prevalence of Strongyloides stercoralis Infection. Pathogens.

[3] Olsen A., van Lieshout L., Marti H., Polderman T., Polman K., Steinmann P., Stothard R., Thybo S., Verweij J.J., Magnussen P. (2009). Strongyloidiasis--the most neglected of the neglected tropical diseases?. Trans. R. Soc. Trop. Med. Hyg..

[4] Buonfrate D., Requena-Mendez A., Angheben A., Muñoz J., Gobbi F., Van Den Ende J., Bisoffi Z. (2013). Severe strongyloidiasis: A systematic review of case reports. BMC Infect Dis..

[5] Corti M. (2016). Strongyloides stercoralis in Immunosuppressed Patients. Arch. Clin. Infect. Dis..

[6] Luvira V., Siripoon T., Phiboonbanakit D., Somsri K., Watthanakulpanich D., Dekumyoy P. (2022). Strongyloides stercoralis: A Neglected but Fatal Parasite. Trop. Med. Infect Dis..

[7] Keiser P.B., Nutman T.B. (2004). Strongyloides stercoralis in the Immunocompromised Population. Clin. Microbiol. Rev..

[8] Czeresnia J.M., Weiss L.M. (2022). Strongyloides stercoralis. Lung.

[9] Ghosh K., Ghosh K. (2007). Strongyloides stercoralis septicaemia following steroid therapy for eosinophilia: Report of three cases. Trans. R. Soc. Trop. Med. Hyg..

[10] Fardet L., Généreau T., Poirot J.-L., Guidet B., Kettaneh A., Cabane J. (2007). Severe strongyloidiasis in corticosteroid-treated patients: Case series and literature review. J. Infect..

[11] Wurtz R., Mirot M., Fronda G., Peters C., Kocka F. (1994). Short report: Gastric infection by Strongyloides stercoralis. Am. J. Trop. Med. Hyg..

[12] Mokhlesi B., Shulzhenko O., Garimella P.S., Kuma L., Monti C. (2004). Pulmonary Strongyloidiasis: The Varied Clinical Presentations. Clin. Pulm. Med..

[13] Mejia R., Nutman T.B. (2012). Screening, prevention, and treatment for hyperinfection syndrome and disseminated infections caused by Strongyloides stercoralis. Curr. Opin. Infect Dis..

[14] Meamar A.R., Rezaian M., Mohraz M., Hadighi R., Kia E.B. (2007). Strongyloides stercoralis hyper-infection syndrome in HIV+/AIDS patients in Iran. Parasitol. Res..

[15] Chordia P., Christopher S., Abraham O.C., Muliyil J., Kang G., Ajjampur S. (2011). Risk factors for acquiring Strongyloides stercoralis infection among patients attending a tertiary hospital in south India. Indian J. Med. Microbiol..

[16] WHO WHO Coronavirus (COVID-19) Dashboard. https://covid19.who.int/.

[17] Covid O., Table S.A. (2021). Ivermectin treatment for Strongyloides infection in patients with COVID-19. Can. Commun. Dis. Rep..

[18] De Wilton A., Nabarro L.E., Godbole G.S., Chiodini P.L., Boyd A., Woods K. (2021). Risk of Strongyloides Hyperinfection Syndrome when prescribing dexamethasone in severe COVID-19. Travel Med. Infect Dis..

[19] Shirley D.A., Moonah S. (2021). COVID-19 and Corticosteroids: Unfamiliar but Potentially Fatal Infections That Can Arise following Short-Course Steroid Treatment. Am. J. Trop. Med. Hyg..

[20] Olivera M.J. (2021). Dexamethasone and COVID-19: Strategies in Low- and Middle-Income Countries to Tackle Steroid-Related Strongyloides Hyperinfection. Am. J. Trop. Med. Hyg..

[21] Boggild A.K., Libman M., Greenaway C., McCarthy A.E. (2016). CATMAT statement on disseminated strongyloidiasis: Prevention, assessment and management guidelines. Can. Commun. Dis. Rep..

[22] Stauffer W.M., Alpern J.D., Walker P.F. (2020). COVID-19 and Dexamethasone: A Potential Strategy to Avoid Steroid-Related Strongyloides Hyperinfection. JAMA.

[23] Rosca E.C., Heneghan C., Spencer E.A., Pluddemann A., Maltoni S., Gandini S., Onakpoya I., Evans D., Conly J.M., Jefferson T. (2023). Coinfection with Strongyloides and SARS-CoV-2: Protocol for a systematic review. medRxiv.

[24] WHO The Use of the WHO-UMC System for Standardised Case Causality Assessment. https://www.who.int/publications/m/item/WHO-causality-assessment.

[25] Alian S., Ahangarkani F., Boskabadi S.J., Kargar-Soleimanabad S., Delavarian L., Pakzad A. (2022). Mucormycosis, one month after recovery from COVID-19: A case report. Ann. Med. Surg..

[26] Babazadeh S., Shokri-Shirvani J., Ranaee M. (2022). Strongyloides Hyperinfection Syndrome Following Corticosteroid Therapy in a Patient with COVID-19 infection: A Case Report. Iran. J. Med. Microbiol..

[27] Feria L., Torrado M., Anton-Vazquez V. (2022). Reactivation of Strongyloides stercoralis in patients with SARS-CoV-2 pneumonia receiving dexamethasone. Med. Clin..

[28] Gautam D., Gupta A., Meher A., Siddiqui F., Singhai A. (2021). Corticosteroids in Covid-19 pandemic have the potential to unearth hidden burden of strongyloidiasis. IDCases.

[29] Kim J.M., Sivasubramanian G. (2022). Strongyloides Hyperinfection Syndrome among COVID-19 Patients Treated with Corticosteroids. Emerg. Infect Dis..

[30] Lier A.J., Davis M.W., Topal J.E. (2020). Antimicrobial Management of Disseminated Strongyloidiasis in a COVID-19 Patient. Am. J. Trop. Med. Hyg..

[31] Lorenzo H., Carbonell C., Vicente Santiago M.B., López-Bernus A., Pendones Ulerio J., Muñoz Bellido J.L., Muro A., Belhassen-García M. (2022). Influence of the drugs used in migrant patients with severe acute respiratory syndrome coronavirus 2 and the development of symptomatic strongyloidiasis. Trans. R. Soc. Trop. Med. Hyg..

[32] Marchese V., Crosato V., Gulletta M., Castelnuovo F., Cristini G., Matteelli A., Castelli F. (2021). Strongyloides infection manifested during immunosuppressive therapy for SARS-CoV-2 pneumonia. Infection.

[33] Babazadeh S., Shokri-Shirvani J., Ranaee M. (2021). Strongyloides stercoralis prophylaxis with ivermectin in COVID-19 patients. Eur. J. Hosp. Pharmacy. Sci. Pract..

[34] Nakandakari Gomez M.D., Marín Macedo H., Seminario Vilca R. (2021). IgA (Henoch Schönlein Purpura) Vasculitis In A Pediatric Patient With COVID-19 And Strongyloidiasis. Rev. Fac. Med. Hum..

[35] Núñez-Gómez L., Comeche B., Subirats M. (2021). Strongyloidiasis: An Important Coinfection in the COVID-19 Era. Am. J. Trop. Med. Hyg..

[36] O’Dowling A., Gillis A. (2022). Strongyloides Hyperinfection Syndrome in a Patient with Asymptomatic COVID-19 Infection. Ir. Med. J..

[37] Patel A., Bender W., Gonzalez E., Williamson M. (2021). A case of disseminated strongyloidiasis during treatment for COVID-19. Chest.

[38] Pintos-Pascual I., López-Dosil M., Castillo-Núñez C., Múñez-Rubio E. (2021). Eosinophilia and abdominal pain after severe pneumonia due to COVID 19. Enferm. Infecc. Microbiol. Clin..

[39] Singh S., Singh U.S. (2022). Coinfection with Strongyloides and Ascaris in a COVID-19-positive male presenting with acute abdomen: A case report. Future Microbiol..

[40] Stylemans D., Van Cauwelaert S., D’Haenens A., Slabbynck H. (2021). COVID-19-Associated Eosinopenia in a Patient With Chronic Eosinophilia Due to Chronic Strongyloidiasis. Infect Dis. Clin. Pract. (Baltim Md).

[41] Nucci M., Portugal R., Pulcheri W., Spector N., Ferreira S.B., de Castro M.B., Noe R., de Oliveira H.P. (1995). Strongyloidiasis in patients with hematologic malignancies. Clin. Infect Dis..

[42] Brigandi R.A., Rotman H.L., Leon O., Nolan T.J., Schad G.A., Abraham D. (1998). Strongyloides stercoralis host-adapted third-stage larvae are the target of eosinophil-associated immune-mediated killing in mice. J. Parasitol..

[43] Genta R.M. (1992). Dysregulation of strongyloidiasis: A new hypothesis. Clin. Microbiol. Rev..

[44] Corti M., Villafañe M.F., Trione N., Risso D., Abuín J.C., Palmieri O. (2011). [Infection due to Strongyloides stercoralis: Epidemiological, clinical, diagnosis findings and outcome in 30 patients]. Rev. Chil. Infectol..

[45] Tanaka M., Hirabayashi Y., Gatanaga H., Aizawa S., Hachiya A., Takahashi Y., Tashiro E., Kohsaka T., Oyamada M., Ida S. (1999). Reduction in interleukin-2-producing cells but not Th1 to Th2 shift in moderate and advanced stages of human immunodeficiency virus type-1-infection: Direct analysis of intracellular cytokine concentrations in CD4+ CD8− T cells. Scand. J. Immunol..

[46] Montes M., Sanchez C., Verdonck K., Lake J.E., Gonzalez E., Lopez G., Terashima A., Nolan T., Lewis D.E., Gotuzzo E. (2009). Regulatory T cell expansion in HTLV-1 and strongyloidiasis co-infection is associated with reduced IL-5 responses to Strongyloides stercoralis antigen. PLoS Negl. Trop. Dis..

[47] Porto A.F., Neva F.A., Bittencourt H., Lisboa W., Thompson R., Alcântara L., Carvalho E.M. (2001). HTLV-1 decreases Th2 type of immune response in patients with strongyloidiasis. Parasite Immunol..

[48] Grove D.I. (1996). Human strongyloidiasis. Adv. Parasitol..

[49] Xie G., Ding F., Han L., Yin D., Lu H., Zhang M. (2021). The role of peripheral blood eosinophil counts in COVID-19 patients. Allergy.

[50] Cortés-Vieyra R., Gutiérrez-Castellanos S., Álvarez-Aguilar C., Baizabal-Aguirre V.M., Nuñez-Anita R.E., Rocha-López A.G., Gómez-García A. (2021). Behavior of Eosinophil Counts in Recovered and Deceased COVID-19 Patients over the Course of the Disease. Viruses.

[51] Padigel U.M., Hess J.A., Lee J.J., Lok J.B., Nolan T.J., Schad G.A., Abraham D. (2007). Eosinophils act as antigen-presenting cells to induce immunity to Strongyloides stercoralis in mice. J. Infect Dis..

[52] Galioto A.M., Hess J.A., Nolan T.J., Schad G.A., Lee J.J., Abraham D. (2006). Role of eosinophils and neutrophils in innate and adaptive protective immunity to larval strongyloides stercoralis in mice. Infect Immun..

[53] Geri G., Rabbat A., Mayaux J., Zafrani L., Chalumeau-Lemoine L., Guidet B., Azoulay E., Pène F. (2015). Strongyloides stercoralis hyperinfection syndrome: A case series and a review of the literature. Infection.

[54] Adedayo O., Grell G., Bellot P. (2002). Hyperinfective strongyloidiasis in the medical ward: Review of 27 cases in 5 years. South Med. J..

[55] Litachevsky V., Peretz S., Schwartz I. (2013). [Clinical-pathologic conference (cpc)—Pulmonary insufficiency in Myasthenia Gravis patient]. Harefuah.

[56] Ribeiro L.C., Rodrigues Junior E.N., Silva M.D., Takiuchi A., Fontes C.J. (2005). [Purpura in patient with disseminated strongiloidiasis]. Rev. Soc. Bras. Med. Trop..

[57] Requena-Méndez A., Buonfrate D., Gomez-Junyent J., Zammarchi L., Bisoffi Z., Muñoz J. (2017). Evidence-Based Guidelines for Screening and Management of Strongyloidiasis in Non-Endemic Countries. Am. J. Trop. Med. Hyg..

[58] Brotherton H., Usuf E., Nadjm B., Forrest K., Bojang K., Samateh A.L., Bittaye M., Roberts C.A., d’Alessandro U., Roca A. (2020). Dexamethasone for COVID-19: Data needed from randomised clinical trials in Africa. Lancet Glob. Health.

[59] Rodríguez-Guardado A., Álvarez-Martínez M.J., Flores M.D., Sulleiro E., Torrús-Tendero D., Velasco M., Membrillo F.J. (2022). Screening for strongyloidiasis in Spain in the context of the SARS-CoV-2 pandemic: Results of a survey on diagnosis and treatment. Enferm. Infecc. Microbiol. Clin..

