# Supplementary material for: Coinfection with Strongyloides and SARS-CoV-2: A Systematic Review
Source: Trop Med Infect Dis. 2023 Apr 25;8(5):248. doi: 10.3390/tropicalmed8050248 (PMC10224069; doi:10.3390/tropicalmed8050248)
Supplement: Supplementary file 1 [file tropicalmed-08-00248-s001.zip › S1. List of excluded studies.pdf]

S1. List of excluded studies

1. Abdoli A, Falahi S, Kenarkoohi A. COVID-19-associated opportunistic infections: a snapshot on the current reports. Clin Exp Med. 2022 Aug;22(3):327-346. doi: 10.1007/s10238-021-00751-7. – review
2. COVID-19 Update: NIH Recommends Against Ivermectin. The Medical Letter on Drugs and Therapeutics 2022. https://secure.medicalletter.org/system/files/private/TML-article-1652e.pdf (Accessed on 2 February 2023)
3. Barkati S, Greenaway C, Libman MD. Strongyloidiasis in immunocompromised migrants to non-endemic countries in the era of COVID-19: what is the role for presumptive ivermectin? J Travel Med. 2022 Jan 17;29(1):taab155. doi: 10.1093/jtm/taab155 – editorial
4. Bradbury RS, Piedrafita D, Greenhill A, Mahanty S. Will helminth co-infection modulate COVID-19 severity in endemic regions? Nat Rev Immunol. 2020 Jun;20(6):342. doi: 10.1038/s41577-020-0330-5. – comment
5. Cai P, Mu Y, McManus DP. The Fight Against Severe COVID-19: Can Parasitic Worms Contribute? Front Immunol. 2022 Feb 11;13:849465. doi: 10.3389/fimmu.2022.849465. – opinion
6. Chacin-Bonilla L, Chacón-Fonseca N, Rodriguez-Morales AJ. Emerging issues in COVID-19 vaccination in tropical areas: Impact of the immune response against helminths in endemic areas. Travel Med Infect Dis. 2021 Jul-Aug;42:102087. doi: 10.1016/j.tmaid.2021.102087. – editorial
7. Covid- O, Table SA; Drugs; Biologics Clinical Practice Guidelines Working Group*. Ivermectin treatment for Strongyloides infection in patients with COVID-19. Can Commun Dis Rep. 2021 Jul 8;47(7-8):316-321. doi: 10.14745/ccdr.v47i78a04. – recommendation
8. De Wilton A, Nabarro LE, Godbole GS, Chiodini PL, Boyd A, Woods K. Risk of Strongyloides Hyperinfection Syndrome when prescribing dexamethasone in severe COVID-19. Travel Med Infect Dis. 2021 Mar-Apr;40:101981. doi: 10.1016/j.tmaid.2021.101981. – opinion
9. Egwang TG, Owalla TJ, Kemigisha M. COVID-19 vaccine trials must include helminth-infected cohorts. Nat Immunol. 2022 Feb;23(2):148. doi: 10.1038/s41590-021-01116-8. – opinion
10. García-Bernalt Diego J, Fernández-Soto P, Márquez-Sánchez S, Santos Santos D, Febrer-Sendra B, Crego-Vicente B, Muñoz-Bellido JL, Belhassen-García M, Corchado Rodríguez JM, Muro A. SMART-LAMP: A Smartphone-Operated Handheld Device for Real-Time Colorimetric Point-of-Care Diagnosis of Infectious Diseases via Loop-Mediated Isothermal

Amplification. Biosensors (Basel). 2022 Jun 16;12(6):424. doi: 10.3390/bios12060424. – preclinical

11. Hays R, Pierce D, Giacomin P, Loukas A, Bourke P, McDermott R. Helminth coinfection and COVID-19: An alternate hypothesis. PLoS Negl Trop Dis. 2020 Aug 17;14(8):e0008628. doi: 10.1371/journal.pntd.0008628. – opinion
12. Jenks NP, Driscoll B, Locke T. Strongyloidiasis Hyperinfection Syndrome in COVID-19 Positive Migrants Treated with Corticosteroids. J Immigr Minor Health. 2022 Dec;24(6):1431-1434. doi: 10.1007/s10903-022-01386-w. – not coinfection
13. Kow CS, Hasan SS. Concurrent Antibiotic Therapy in Disseminated Strongyloidiasis. Am J Trop Med Hyg. 2020 Nov;103(5):2149. doi: 10.4269/ajtmh.20-1059a- comment
14. Lier AJ, Davis MW, Topal JE. Antimicrobial Management of Disseminated Strongyloidiasis in a COVID-19 Patient. Am J Trop Med Hyg. 2020 Nov;103(5):2150. doi: 10.4269/ajtmh.20-1059b. – comment
15. Markus MB. Helminthiasis, eosinophils, COVID-19 and vaccination. S Afr J Infect Dis. 2022 Jun 29;37(1):423. doi: 10.4102/sajid.v37i1.423. – letter
16. Mewara A, Sahni N, Jain A. Considering opportunistic parasitic infections in COVID-19 policies and recommendations. Trans R Soc Trop Med Hyg. 2021 Nov 1;115(11):1345-1347. doi: 10.1093/trstmh/trab142. – recommendation
17. Meyer A, Regunath H, Rojas-Moreno C, Salzer W, Christensen G. Imported Infections in Rural Mid-West United States - A Report from a Tertiary Care Center. Mo Med. 2020 Mar-Apr;117(2):89-94. – not COVID-19
18. Mohareb AM, Rosenberg JM, Bhattacharyya RP, Kotton CN, Chu JT, Jilg N, Hysell KM, Albin JS, Sen P, Bloom SM, Schiff AE, Zachary KC, Letourneau AR, Kim AY, Hurtado RM. Preventing Infectious Complications of Immunomodulation in COVID-19 in Foreign-Born Patients. J Immigr Minor Health. 2021 Dec;23(6):1343-1347. doi: 10.1007/s10903-021-01225-4. – recommendation
19. Norman FF, Chamorro S, Braojos F, López-Miranda E, Chamorro J, González I, Martín O, Pérez-Molina JA. Strongyloides in bronchoalveolar lavage fluid: practical implications in the COVID-19 era. J Travel Med. 2022 Jan 17;29(1):taab114. doi: 10.1093/jtm/taab114. – not COVID-19
20. Olivera MJ. Dexamethasone and COVID-19: Strategies in Low- and Middle-Income Countries to Tackle Steroid-Related Strongyloides Hyperinfection. Am J Trop Med Hyg. 2021 Mar 15;104(5):1611-1612. doi: 10.4269/ajtmh.20-1085. – perspective
21. Paniz-Mondolfi AE, Ramírez JD, Delgado-Noguera LA, Rodriguez-Morales AJ, Sordillo EM. COVID-19 and helminth infection: Beyond the Th1/Th2 paradigm. PLoS Negl Trop Dis. 2021 May 24;15(5):e0009402. doi: 10.1371/journal.pntd.0009402. – opinion

22. Páramo-Zunzunegui J, Rubio-López L, Benito-Barbero S, Muñoz-Fernández Á. Eosinophilic appendicitis due to Strongyloides stercoralis: a challenging differential diagnosis for clinicians. BMJ Case Rep. 2021 Jun 1;14(6):e239685. doi: 10.1136/bcr-2020-239685. – not COVID-19
23. Pereira CVM, Mastandrea GRA, Medeiros ACCS, Gryschek RCB, Paula FM, Corral MA. COVID-19 and strongyloidiasis: what to expect from this coinfection? Clinics (Sao Paulo). 2021 Nov 19;76:e3528. doi: 10.6061/clinics/2021/e3528. – opinion
24. Pivoto João GA, Alves Antunes IF, Mendes Dos Santos L. Fatal Disseminated Strongyloidiasis with Periumbilical Purpura. Am J Trop Med Hyg. 2021 Jun 28;105(4):860-861. doi: 10.4269/ajtmh.21-0464. – not COVID-19
25. Rodríguez-Guardado A, Álvarez-Martínez MJ, Flores MD, Sulleiro E, Torrús-Tendero D, Velasco M, Membrillo FJ; En Nombre Del Grupo De Estudio De Patología Importada De La Seimc. Cribado de estrongiloidiasis en España en el contexto de la pandemia SARS-CoV-2: resultados de una encuesta sobre diagnóstico y tratamiento [Screening for strongyloidiasis in Spain in the context of the SARS-CoV-2 pandemic: results of a survey on diagnosis and treatment]. Enferm Infecc Microbiol Clin. 2021 Dec 15. Spanish. doi: 10.1016/j.eimc.2021.11.010. – epidemiological study; no data on coinfection
26. Ruan J, Moskowitz AJ, Mehta-Shah N, et al. Multi-Center Phase II Study of Oral Azacitidine (CC-486) Plus CHOP As Initial Treatment for Peripheral T-Cell Lymphoma (PTCL). Blood 2020; 136 (Supplement 1): 33-34. https://doi.org/10.1182/blood-2020-136023 - lymphoma study
27. Shirley DA, Moonah S. COVID-19 and Corticosteroids: Unfamiliar but Potentially Fatal Infections That Can Arise following Short-Course Steroid Treatment. Am J Trop Med Hyg. 2021 Jan 6;104(3):790-793. doi: 10.4269/ajtmh.20-1471. – perspective
28. Smith CJ, Gaballah AH, Bowers K, Baxter C, Caruso CR. Atypical pulmonary presentation of Strongyloides stercoralis hyperinfection in a patient with philadelphia chromosome-positive acute lymphoblastic leukemia: Case report. IDCases. 2022 Jun 6;29:e01530. doi: 10.1016/j.idcr.2022.e01530. – not coinfection
29. Stauffer WM, Alpern JD, Walker PF. COVID-19 and Dexamethasone: A Potential Strategy to Avoid Steroid-Related Strongyloides Hyperinfection. JAMA. 2020 Aug 18;324(7):623-624. doi: 10.1001/jama.2020.13170. – opinion
30. Toor J, Adams ER, Aliee M, et al. Predicted Impact of COVID-19 on Neglected Tropical Disease Programs and the Opportunity for Innovation. Clin Infect Dis. 2021 Apr 26;72(8):1463-1466. doi: 10.1093/cid/ciaa933. – viewpoint
31. Turkia M. COVID-19 and Strongyloidiasis (January 18, 2021). Available at SSRN: https://ssrn.com/abstract=3766955 or http://dx.doi.org/10.2139/ssrn.3766955 - viewpoint

32. Vellere I, Graziani L, Tilli M, Mantella A, Campolmi I, Mencarini J, Borchi B, Spinicci M, Antonelli A, Rossolini GM, Bartoloni A, Zammarchi L. Strongyloidiasis in the COVID era: a warning for an implementation of the screening protocol. Infection. 2021 Oct;49(5):1065-1067. doi: 10.1007/s15010-021-01621-w. - opinion
