# Supplementary material for: Coinfection with Strongyloides and SARS-CoV-2: A Systematic Review
Source: Trop Med Infect Dis. 2023 Apr 25;8(5):248. doi: 10.3390/tropicalmed8050248 (PMC10224069; doi:10.3390/tropicalmed8050248)
Supplement: Supplementary file 1 [file tropicalmed-08-00248-s001.zip › S2. List of included studies.pdf]

S2. List of included studies

1. Alian S, Ahangarkani F, Boskabadi SJ, et al. Mucormycosis, one month after recovery from COVID-19: A case report. Ann Med Surg (Lond). 2022; 78:103911. doi: 10.1016/j.amsu.2022.103911.
2. Babazadeh SS, Shokri-Shirvani J, Ranaee M.-SJRM. Strongyloides hyperinfection syndrome following corticosteroid therapy in a patient with covid-19 infection: A case report. Iranian Journal of Medical Microbiology 2022; 16(3):267-270. Doi: 10.30699/ijmm.16.3.267
3. Feria L, Torrado M, Anton-Vazquez V. Reactivation of Strongyloides stercoralis in patients with SARS-CoV-2 pneumonia receiving dexamethasone. Med Clin (Barc). 2022; 11;158(5):242-243. English, Spanish. doi: 10.1016/j.medcli.2021.05.004.
4. Gautam D, Gupta A, Meher A, et al. Corticosteroids in Covid-19 pandemic have the potential to unearth hidden burden of strongyloidiasis. IDCases. 2021; 25:e01192. doi: 10.1016/j.idcr.2021.e01192.
5. Kim JM, Sivasubramanian G. Strongyloides Hyperinfection Syndrome among COVID-19 Patients Treated with Corticosteroids. Emerg Infect Dis. 2022; 28(7):1531-1533. doi: 10.3201/eid2807.220198.
6. Lier AJ, Tuan JJ, Davis MW, et al. Case Report: Disseminated Strongyloidiasis in a Patient with COVID-19. Am J Trop Med Hyg. 2020; 103(4):1590-1592. doi: 10.4269/ajtmh.20-0699.
7. Lorenzo H, Carbonell C, Vicente Santiago MB, et al. Influence of the drugs used in migrant patients with severe acute respiratory syndrome coronavirus 2 and the development of symptomatic strongyloidiasis. Trans R Soc Trop Med Hyg. 2022; 116(5):440-445. doi: 10.1093/trstmh/trab152.
8. Marchese V, Crosato V, Gulletta M, et al. Strongyloides infection manifested during immunosuppressive therapy for SARS-CoV-2 pneumonia. Infection. 2021; 49(3):539-542. doi: 10.1007/s15010-020-01522-4.
9. Martínez Suárez A, Castillejo García R, Castillo Martin C, et al. Strongyloides stercoralis prophylaxis with ivermectin in COVID-19 patients. European Journal of Hospital Pharmacy Science and Practice. 2021/00 2021;28(Suppl 1): A115-A116. doi:10.1136/ejhpharm-2021-eahpconf.237
10. Nakandakari Gomez MD, Marín Macedo H, Seminario Vilca R. IgA (Henoch Schönlein Purpura) Vasculitis In A Pediatric Patient With COVID-19 And Strongyloidiasis. Vasculitis por IgA (Púrpura de Henoch Schönlein) en un paciente pediátrico con COVID-19 y Estrongiloidiasis. Rev Fac Med Hum. 2021/01 2021;21(1):199-205. doi:10.25176/RFMH.V21I1.3265
11. Núñez-Gómez L, Comeche B, Subirats M. Strongyloidiasis: An Important Coinfection in the COVID-19 Era. Am J Trop Med Hyg. 2021; 105(5):1134-1135. doi: 10.4269/ajtmh.21-0677.

12. O'Dowling A, Gillis A. Strongyloides Hyperinfection Syndrome in a Patient with Asymptomatic COVID-19 Infection. Ir Med J. 2022; 115(4):591.
13. Patel A, Bender W, Gonzalez E, Williamson M. A case of disseminated strongyloidiasis during treatment for COVID-19. Chest 2021;160(4): A278-A278. doi:10.1016/j.chest.2021.07.285
14. Pintos-Pascual I, López-Dosil M, Castillo-Núñez C, Múñez-Rubio E. Eosinophilia and abdominal pain after severe pneumonia due to COVID 19. Enferm Infecc Microbiol Clin (Engl Ed). 2021; 39(9):478-480. doi: 10.1016/j.eimce.2021.08.007.
15. Singh S, Singh US. Coinfection with Strongyloides and Ascaris in a COVID-19-positive male presenting with acute abdomen: a case report. Future Microbiol. 2022; 17:1099-1105. doi: 10.2217/fmb-2022-0027.
16. Stylemans D, Van Cauwelaert S, D'Haenens A, Slabbynck H. COVID-19-Associated Eosinopenia in a Patient With Chronic Eosinophilia Due to Chronic Strongyloidiasis. Infect Dis Clin Pract (Baltim Md). 2021; 29(5):e305-e306. doi: 10.1097/IPC.0000000000000991.
